# Wrinkled Nitrogen-doped Carbon Belts

**DOI:** 10.1038/s41598-018-21898-6

**Published:** 2018-02-23

**Authors:** Juan L. Fajardo-Díaz, Florentino López-Urías, Emilio Muñoz-Sandoval

**Affiliations:** Advanced Materials Division, IPICYT, Camino a la Presa San José 2055, San Luis Potosí, 78216 Mexico

## Abstract

Graphene, carbon nanotubes, and fullerenes are nanomaterials with outstanding properties such as electrical, thermal, mechanical strength, flexibility, and high surface area. These nanomaterials are used as building blocks for the construction of novel and astonishing 3D-dimensional networks. In the present work, nitrogen-doped carbon belt (N-CB) structures containing wrinkled carbon fibres as building blocks were synthesized under unstable conditions in a chemical vapour deposition experiment. N-CB structures with 0.2–3.0 microns of wide and 350 nm thick were assembled from complex individual wrinkled carbon fibres grown on Co/Cu films. These complex structures have a tubular appearance, showing holed and wrinkled graphite layers. Sulphur and copper atoms drastically affect the catalytic role of cobalt, changing the conventional growth of carbon nanotubes. Chemical functional groups, N-doping, and carbons hybridizations involved in the winkled carbon fibres are investigated. These findings provides a novel material that can be used as an excellent oxygen-reduction reaction catalyst or nano-electronics component.

## Introduction

The creation of new 2D carbon allotropes^1,2^ or 3D macroscopic architectures^3,4^ is changing the perspective of carbon nanomaterials research. Instead of investigating individual or groups of carbon nanomaterials, hybrid or complex 2D or 3D structures are the focus of many research groups. Carbon nanotube networks^5^, graphene wrinkled sheets^6^, and nanofibres^7^ serve as building blocks for these new 3D carbon nanomaterials. Super capacitors, super hydrophobic materials, exceptional electrodes for lithium batteries and other breakthrough applications have been derived from such complex constructions^8–10^. The possibility of building arrangements, such as multi-walled carbon nanotubes (MWCNTs) networks, and graphene-MWCNT hybrids, MWCNT sponges by different methods, such as chemical vapour deposition (CVD) synthesis, the template method, CNT suspension or hydrothermal reduction is a frequent topic of theoretical and experimental investigations^11–16^. Numerous techniques have been used to create such nanomaterials^3^. For example, nano-building blocks of single walled carbon nanotubes (SWCNTs) and MWCNTs were used as construction components to create macrostructures in a sol-gel solution^17,18^. 3D carbon nanostructures were fabricated using an inorganic or polymeric matrix as a template^19,20^.

It has been demonstrated that the structural and physical-chemical properties of such carbon nanomaterials depend on the synthesis conditions, precursors and catalyst^21–23^. However, the use of bimetallic catalysts to grow 3D carbon architectures is far from understood^24^. Chambers *et al*.^25^ reported a multi-growth arrangement of carbon nanotubes on specific crystallographic planes of cobalt nanoparticles as consequence of adding copper during the synthesis. Other mixtures, such as Ag-Co, Fe-Co, and Ni-Cu catalysts, favoured carbon nanostructures with unusual morphologies^26,27^. Doping is another important issue to consider when producing carbon 3D frameworks. The presence of large amounts of sp^3^ hybridization, can be obtained by different dopants, such as oxygen^28,29^, boron^30,31^, phosphorous^32,33^, nitrogen^34^ and sulphur^35,36^. In general, sulphur acts as an anchoring point for the incorporation of other species-producing the formation of branches or the formation of negative curvatures over carbon nanotubes. Sulphur also promotes changes in the electrical properties of the complicated 3D MWCNTs framework^20,35–38^. Carbon morphologies, such as wrinkled graphene and wrinkled N-CNTs, can be used to fabricate complex 3D architectures without the participation of foreign atoms^39^. Chen *et al*.^40^ modified the structure of graphene layers by mechanical processes, achieving the formation of wrinkled graphene with super-hydrophobicity properties. In the present investigation, assembled wrinkled carbon fibres forming belts were produced using aerosol-assisted chemical vapour deposition (AACVD)-based template synthesis. Based on different characterization techniques, evidence is provided regarding the morphology, graphitization degree, nitrogen doping, chemical groups, and structural irregularities of these fibres.

## Results

**Figure 1:**
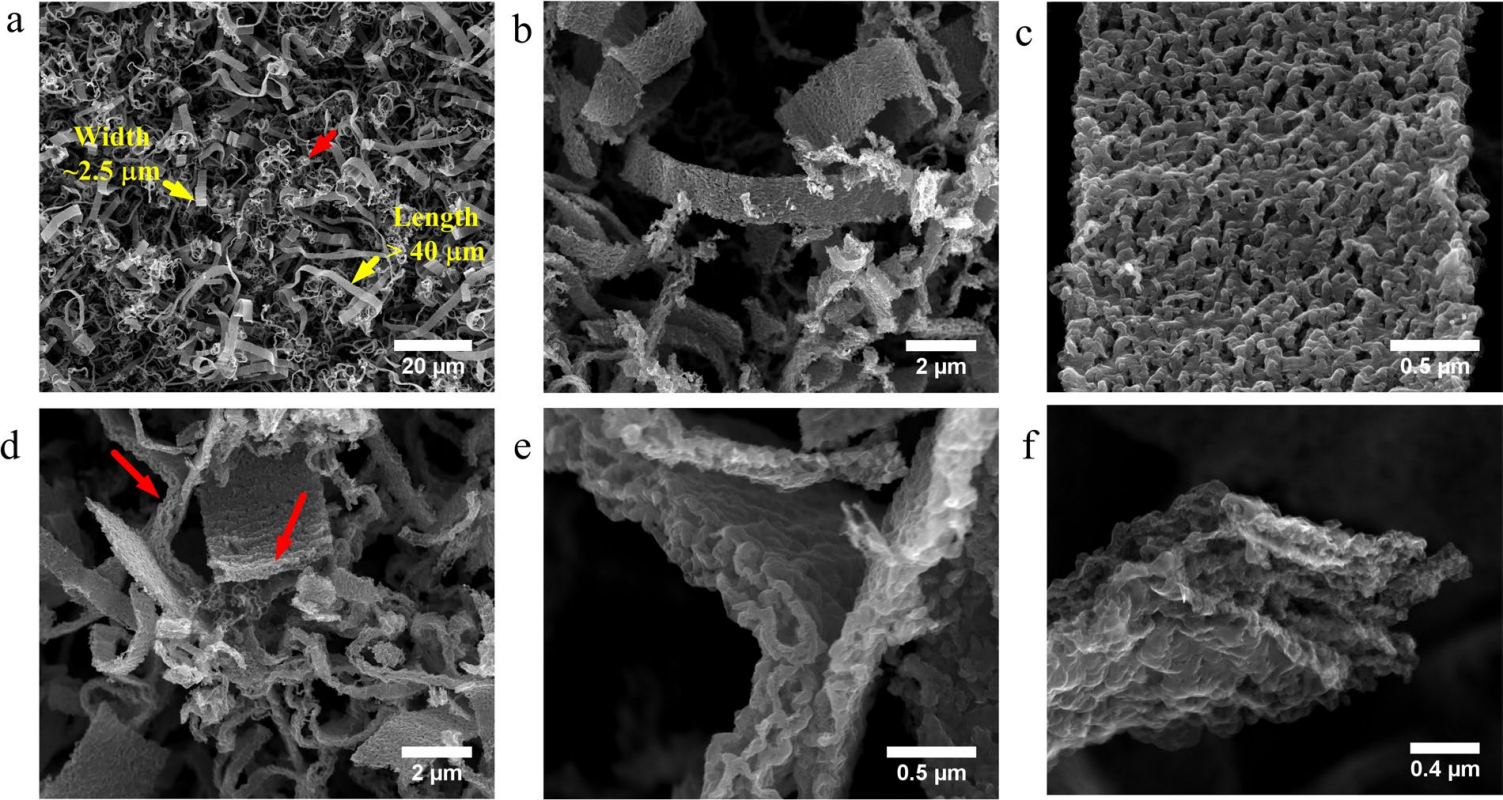
SEM images of the synthesized nitrogen-doped carbon belt (N-CB) structures. (**a**) Low magnification image showing the synthesized sample. (**b**) N-CB of ~2 μm width and 5–20 μm length. (**c**) Magnified image showing the N-CB. (**d**) Front view of N-CBs showing the side and front views (see the arrows). (**e**) Cross section view of a N-CB, and (**f**) the tip structure of a N-CB.

First, Fig. 1 shows different views of the N-CB structures. The low magnification SEM image (Fig. 1a) clearly shows that two predominate structures were found in the sample, formed by N-CBs (see yellow arrows) and wrinkled carbon fibres (see the red arrow) that was several microns in length (10–50 μm). The N-CBs had widths of ~2.5 μm, with a 350 nm of thickness. Close SEM images of the sample revealed that the N-CBs had a porous texture and corrugated surface, as shown in Fig. 1(b,c). The lateral and front views of the N-CB structures can be seen in Fig. 1(d–f). Further, SEM images showing N-CBs can be seen in Fig. S2 (Supplementary Information). In order to define a wavelength of wrinkles of the N-CB, the Fig. S2g was slightly modified by an image processing program (imageJ). After the FFT analysis, the superior inset included in this figure was obtained. The resultant wavelength was 0.03 μm that correspond to the diameter of individual wrinkled carbon fibres. If instead of the FFT analysis a surface representation is used, the inferior inset of Fig. S2g is derived. In this case, a wavelength of 0.75 μm is obtained that correspond to the distance between two maxima (see inferior inset in Fig. S2g).

SEM images and the corresponding backscattering mode revealed the presence of metal-nanoparticles in the N-CBs (Fig. S3 in Supplementary Information). Figure S3(a,b) shows that the tip of the N-CBs contained a bar-shape metal nanoparticle and was composed of copper-oxide hosting cobalt nanoparticles of different sizes. Small copper nanoparticles distributed over the N-CB structure were also observed as is shown in Fig. S3(e,f). Both situations were revealed by EDS-mapping (Fig. S4 in the Supplementary Information).

**Figure 2:**
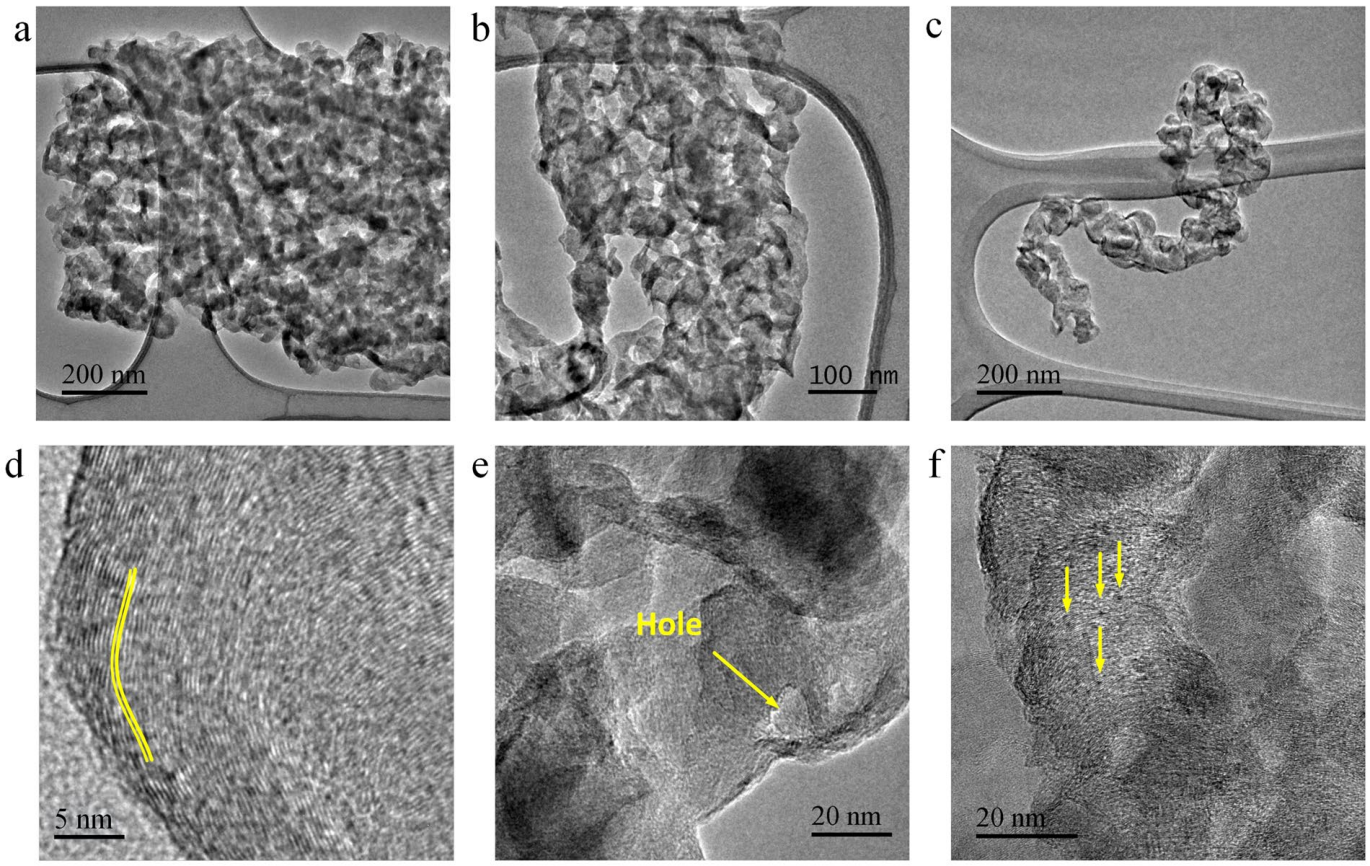
TEM and HRTEM images of N-CB structures. (**a,b**) Top views of N-CBs. The arrows in (**b**) indicate the wrinkled carbon fibres. (**c**) High magnification image of a wrinkled carbon fibre. (**d–f**) HRTEM images of wrinkled carbon fibres. (**d**) Curved graphite layers with an interlayer distance of ~3.5 Å, (**e**) Holed wrinkled carbon fibres, and (**f**) Cu-nanoparticles of ~2 nm diameter anchored on the surface of wrinkled carbon fibre.

TEM images showed the wrinkled corrugated N-CB structures (Fig. 2). A single N-CB structure with an 800 nm width, irregular edges and wrinkled morphology was clearly observed (Fig. 2a). Convincing evidence regarding the internal structure of the N-CBs is found in Fig. 2b, which clearly shows that the N-CBs were formed by assembled wrinkled carbon fibres, as indicated by the arrows. Due to the irregular junctions between the carbon fibres, holes were created (Fig. 2b). An individual wrinkled carbon fibre is shown in Fig. 2c. In Fig. 2d, a HRTEM image of the carbon fibre is shown, revealing that its structure consists of a curved graphite material with an estimated interlayer distance of ~3.5 Å. The presence of holes in the carbon fibre is shown in Fig. 2e. Small metal nanoparticles that are 2–5 nm in diameter anchored to the surface of the carbon fibre are shown in Fig. 2f. EDS analysis revealed that these small nanoparticles are mainly composed of Cu (Fig. S4). Further, TEM images showing the complex structure of different carbon fibres are displayed in Fig. S5(g–i) where hollows and stacked graphitic layers are observed (see yellow and red arrows).

**Figure 3:**
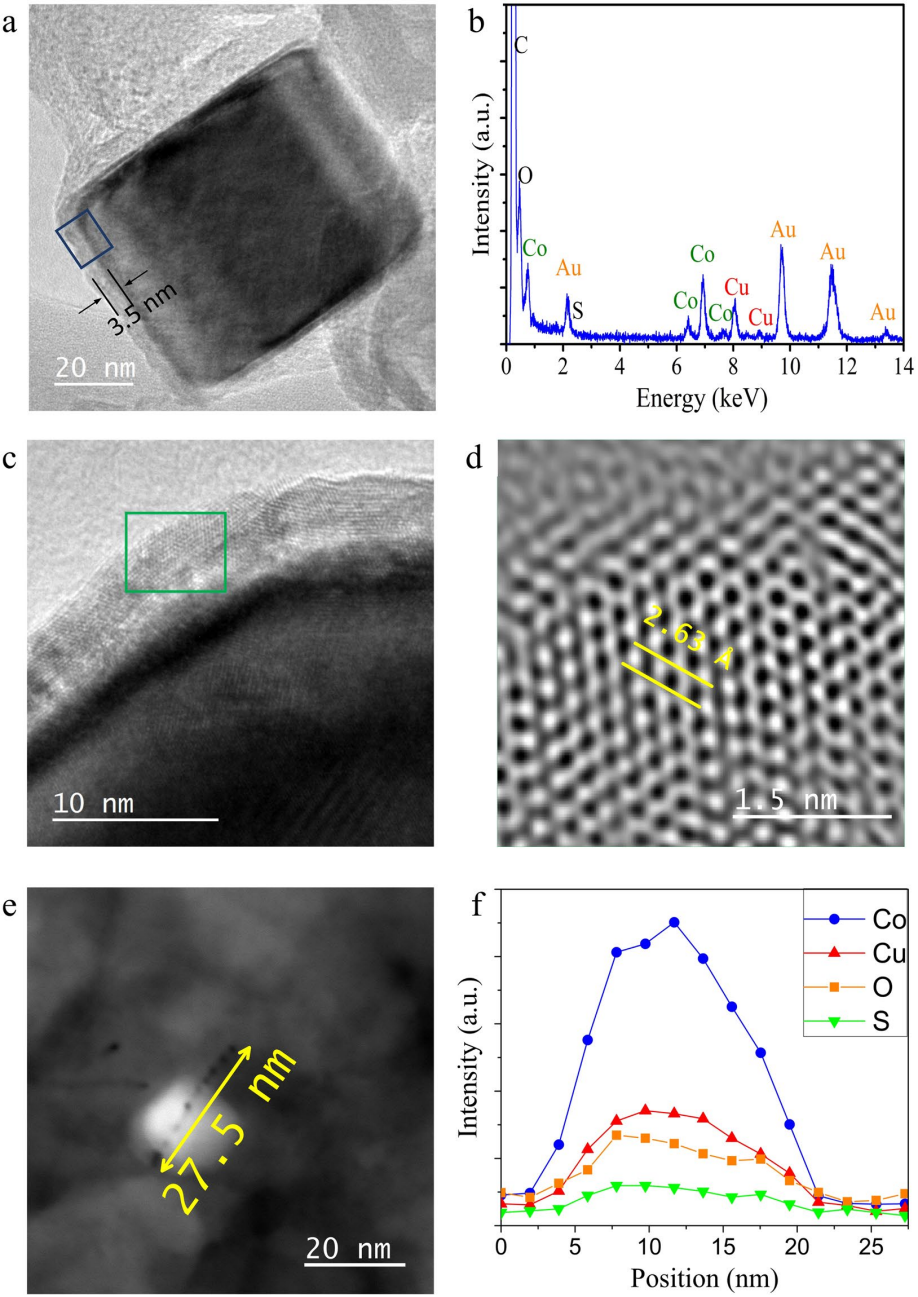
(**a**) HRTEM images of a catalytic Co-nanoparticle. (**b**) EDS analysis revealing the presence of Co, Cu, and O in the catalytic particle. (**c**) Few layers of copper oxide surrounding the Co nanoparticle. (**d**) FFT analysis on the region enclosed in (**c**) yielded an intelayer distance of 2.63 Å that correspond to the (012) crystallographic plane of cobalt-copper oxide. (**e**) Z-contrast TEM image of a Co nanoparticle of ~20 nm diameter and the corresponding elemental line-scan along the nanoparticle (**f**) revealing Co, Cu, O, and S.

**Figure 4:**
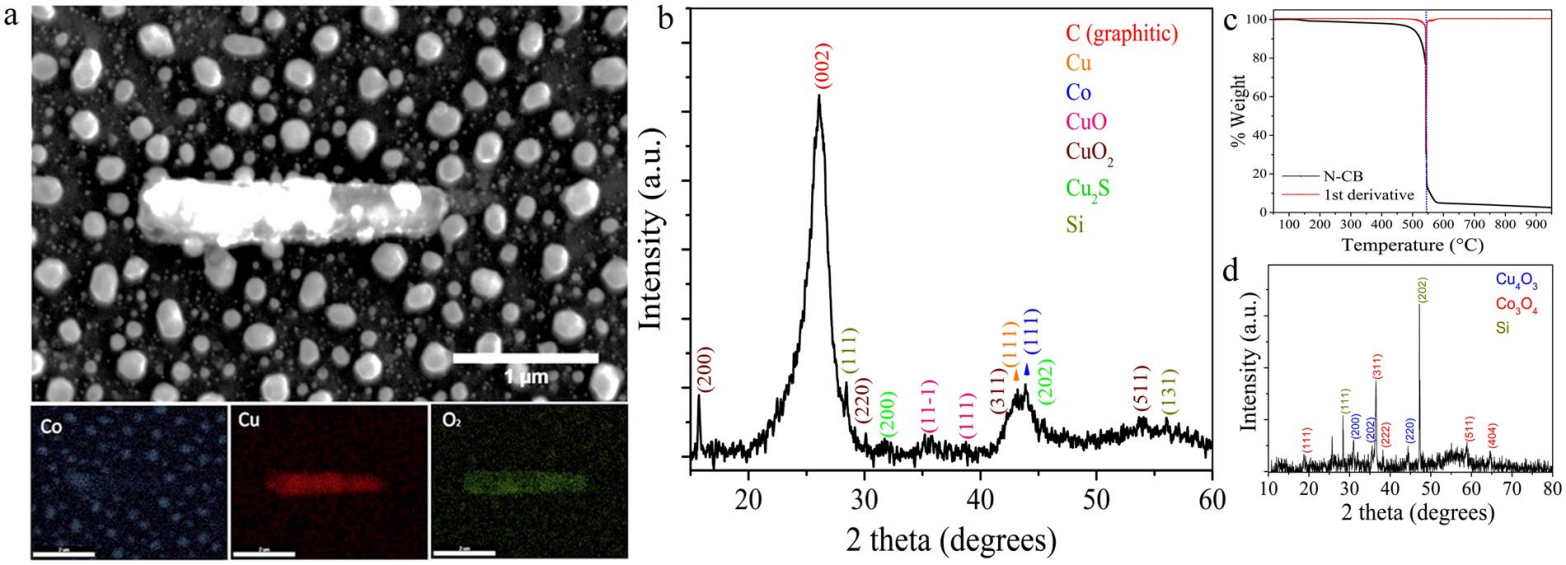
(**a**) SEM image of Co/Cu film exposed to heat-treatment during 2 hours in the absence of precursors. The formation of bar-shaped particles and semi-spherical are observed; the EDS-mapping analysis of cobalt (blue), copper (red) and oxygen (green) are also showed. (**b**) XRD pattern of N-CBs showing the presence graphite material including Co (FCC), Cu (FCC), CuO, CuO_2_, Cu_2_S, and Si. (**c**) TGA with an oxidation temperature of 546 °C and the remaining weight of 2.5% at 950 °C. (**d**) XRD patterns of the TGA remaining material revealing the presence of Co_3_O_4_, Cu_2_O, and Si.

HRTEM characterizations of the catalytic particles responsible for carbon fibre formation are shown in Fig. 3. EDS analysis demonstrated that the nanoparticle is mainly composed by Co. The carbon fibre preferentially grew on of the faces of the cobalt particles (Fig. 3a). Chambers *et al*.^25^ reported that carbon fibres grow preferentially perpendicular to the face defined by the (111) plane of Co nanoparticles. Furthermore, the cobalt particle was surrounded by a thin layer of ~3.5 nm. EDS analysis performed on the thin layer (see blue square) revealed the presence of Co, Cu and O (Fig. 3b). A high magnification image, allowing for better identification of the structure of the thin layer (Fig. 3c), and a fast Fourier transform (FFT) refinement was performed over the green square, revealing an interlayer distance of ~2.63 Å that corresponds to the (012) crystallography planes of CuO (Fig. 3d). Figure 3e shows a Z-contrast TEM image of a Co nanoparticle with a ~20 nm diameter. Elemental line-scan along the nanoparticle revealed the presence of Co, Cu, O, and S (Fig. 3f). Notice that the main contribution comes is Co element, followed by Cu and O with traces of S. The TEM observations suggest that carbon fibres grow on Co nanoparticles, likely during the synthesis. Sulfur and copper-oxide hinder the growth of carbon nanotubes, forming defected carbon fibres. Assembled defective carbon fibre formation occurs when the cobalt nanoparticles described above come together, as shown in Fig. S3(c,d). The assembled carbon fibres adopt a belt morphology when cobalt nanoparticles are hosted on the surface of the copper-oxide bar-shape structures (Fig. 4a).

Figure 4a exhibits the EDS-mapping analysis of Co/Cu film exposed to heat-treatment during 2 hours in absence of precursors under an Ar-H_2_ flow. There are two type of nanostructures obtained after this procedure: copper-oxide bar-shaped particles covered by semi-spherical copper nanoparticles ~80 nm attached and cobalt spherical particles. XRD characterizations of the N-CBs revealed the presence of different crystalline phases (Fig. 4b). The peak at 26.4° corresponds to the (002) crystallographic plane of graphite materials with an interlayer distance of ~3.4 Å. The Co and Cu signals in a face-centred cubic structure were identified as COD 4313211 and COD 9011619, respectively, and preferentially oriented in the (111) crystallographic plane. Copper oxide (II), copper sulphide (I) and copper peroxide (II) (COD 1011148, COD 9005550 and COD 1521320, respectively) were also identified. The presence of Si is from the Si/SiO_2_ substrate. The TGA results for N-CBs are shown in Fig. 4c. The sample lost 1% of its weight at 150 °C due to the removal of oxygen functional groups or water evaporation. At 500 °C, a weight loss of ~2% was reached. This behaviour is related to the oxidation of amorphous carbon. Finally, 97.5% of the weight was lost at 545 °C. This fact indicates that the N-CBs are thermally stable with. The sharp oxidation showed in the TGA curve could be related to the fact that N-CBs are graphitic structures with several edges and defects. N-CBs are thermally stable with an oxidation temperature that is higher than that obtained for nitrogen-doped carbon nanotube that is 430 °C^33^. The residual or remaining material at 950 °C was 2.5% of the initial total weight of the sample, revealing the presence of Cu_4_O_3_, Co_3_O_4_, and Si by XRD (Fig. 4d).

**Figure 5:**
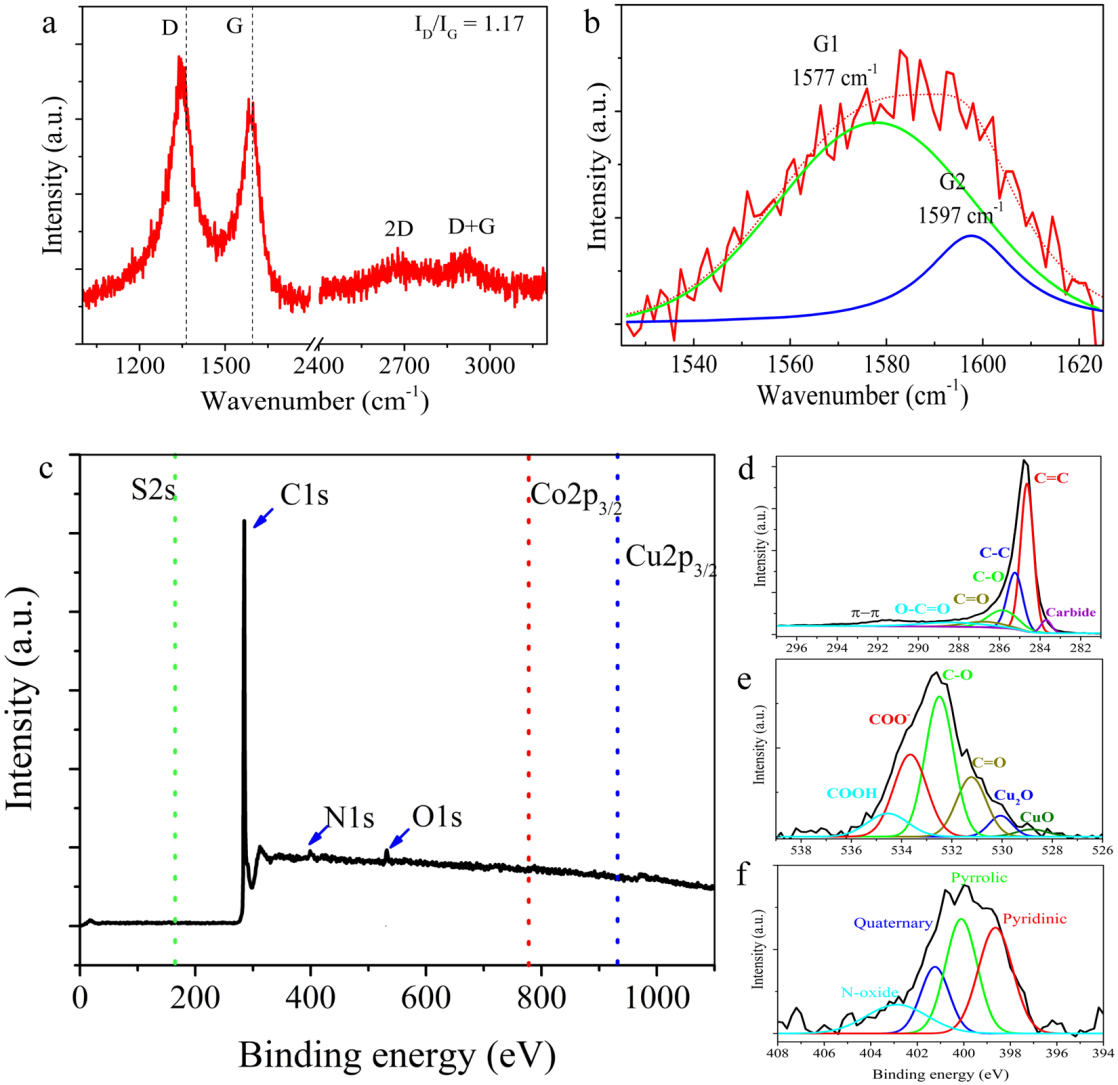
(**a**) Raman shift of N-CBs. The intensity ratio of the D-Raman and G-Raman peaks (I_D_/I_G_) are indicated. (**b**) Deconvolution analysis of the G-Raman peak showing the presence of two peaks (G1 peak placed at 1577 cm^−1^ and G2 peak at 1597 cm^−1^). (**c**) XPS survey scans revealing N, C, O, and S. (**d–f**) Deconvoluted XPS spectra. (**d**) C1s spectra indicating sp^2^ hybridized carbons (C=C), sp^3^ hybridized carbons (C-C), and C=O, C-O, COO^−^, and metal carbide compounds. (**e**) O1s spectra show Cu(II), Cu(I) oxide, COO^−^, C=O, and C-O bonds. (**f**) N1s spectra present the N-pyridinic, N-pyrrolic, N-quaternary, and pyridine-oxide. Binding energies are indicated in Table S1 (Supplementary Information).

Raman spectroscopy characterization is depicted in Fig. 5a. The typical peaks of graphite materials with a D-band peak located at 1345 cm^−1^ are identified, which are related to defects (vacancies, edges, and corrugation). The G-band peak was observed at 1580 cm^−1^; the presence of the overtone 2D-band peak at 2677 cm^−1^ and D + G peak at 2916 cm^−1^ are related to the formation of a few layers of graphite. Deconvolution analysis of the G-band peak (Fig. 5b) revealed the presence of two peaks, G1 and G2 centred at 1577 cm^−1^ and 1597 cm^−1^, respectively. The G1-band peak is attributed to the in-plane vibration of the C-C bonds; however, the G2 peak is due to defective graphite layers (edges, N-doping, and vacancies)^41–43^. G-band splitting was also observed in multi-walled carbon nanotubes and few-layered graphene. In multi-walled carbon nanotubes, the two-peak decomposition of the G-band Raman peak was related to the innermost and outermost layers^44^, where the displacement to the right was affected by functionalities on the outermost layer in MWCNTs. In few-layered graphene, G-band splitting was attributed to *p*-type doping^45^. The estimated intensity ratio of the D-band and G-band peaks (I_D_/I_G_) was 1.17, indicating the presence of defects.

XPS measurements were conducted for surface elemental analysis of the N-CBs samples. The presence of carbon, nitrogen, and oxygen is clearly shown in Fig. 5c. Deconvolution analysis clearly identified the binding energies of the bonds and chemical functional groups. The full width at half maximum (FWHM), peak gravity centre, and integrated area under each curve are shown in Table S1. Figure 5(d–f) depicts the expanded XPS spectra for C1s, O1s, and N1s, respectively. For the C1s peak, sp^2^ and sp^3^ hybridized carbon and oxidized carbon species, such as C-O, C=O, and COO^−^_,_ were attributed to the presence of phenolic, carbonyl, carboxyl, and epoxy groups (Table S1). These carbon-oxygen bonds could also be part of more complex systems such as ethoxy, ether, and ester groups attached to the N-CB surface. The expanded O1s peak revealed the presence of C-O, C=O, COO^−^, and a metal oxide compound (Fig. 5e). The C-O bonds were associated with phenolic groups on the surface or incorporation of oxygen in an ester chemical group. The O-C=O species were attributed to the presence of ester or carboxyl groups. The expanded N1s peak revealed the presence of N-pyrrolic, N-pyridinic, N-quaternary, and N-O bonds. A quantitative estimation from the deconvolution analysis showed that N-pyrrolic and N-pyridinic doping dominated in the sample at 33.7% and 32.6%, respectively. These types of doping are in graphite layers in the presence of defects, such as vacancies and edges. The N-pyrrolic doping in the N-CBs is important for chemical reaction applications. Previous investigations demonstrated that wrinkled graphene sheets doped with N-pyrrolic are highly efficient for oxygen-reduction reaction (ORR) catalyst activity^46,47^. Furthermore, N-pyridinic or N-substitutional doping introduces electrons into the systems, but does not produce considerable alterations to the hexagonal structure. These both features are important for electronic device applications^48,49^.

Figure S6 shows three important physicochemical properties of N-CB structures grown over the Si substrate (Fig. S6a). For example, one important feature of the N-CB is the hydrophobicity (Fig. S6b) that can be applied in membranes distillation process. Also, N-CB electrode shows a higher ORR current than a graphite electrode (Fig. S6c). Due to the Co nanoparticle morphologies on the substrate-N-CB system, they exhibit superparamagnetic behaviour (Fig. S6d) which is important in cell tracking applications. To investigate the role of precursors in the AACVD experiment, the Cu/Co films were exposed to the following: (a) 100% w/w ethanol; (b) 100% w/w benzylamine; (c) 49.90% w/w benzylamine and 50.09% w/w ethanol; (d) 99.8% w/w ethanol and 0.12% w/w thiophene; (e) 99.4% w/w benzylamine and 0.51% w/w thiophene, and f) 49.75% w/w benzylamine, 49.93% w/w ethanol and 0.32% w/w thiophene. The cases shown above reveal interesting trends depending of the type of carbon structure is synthetized (Fig. S7). Figure S7(a–c) refer to samples synthesized without thiophene and Fig. S7(d–f) refer to those synthesized with thiophene. It is clear that the thiophene and benzylamine favored the formation of wrinkled carbon fibres. When benzylamine and thiophene are used as precursors, the structures are similar to the wrinkled carbon fibres of N-CBs (Fig. S7e). In the absence of Cu and thiophene, the N-CBs are no longer formed. By mapping the reactor in the AACVD experiments, it was determined that N-CBs grew only in the intermediate where the two furnaces were joined. Moreover, a well-defined belt structure occurred after 2 hours of growth. Figure S7(g–l) shows the different synthetized structures when the concentration of cobalt and copper is varied on the Si substrate. In these cases the yield of N-CBs is increased.

## Discussion

**Figure 6:**
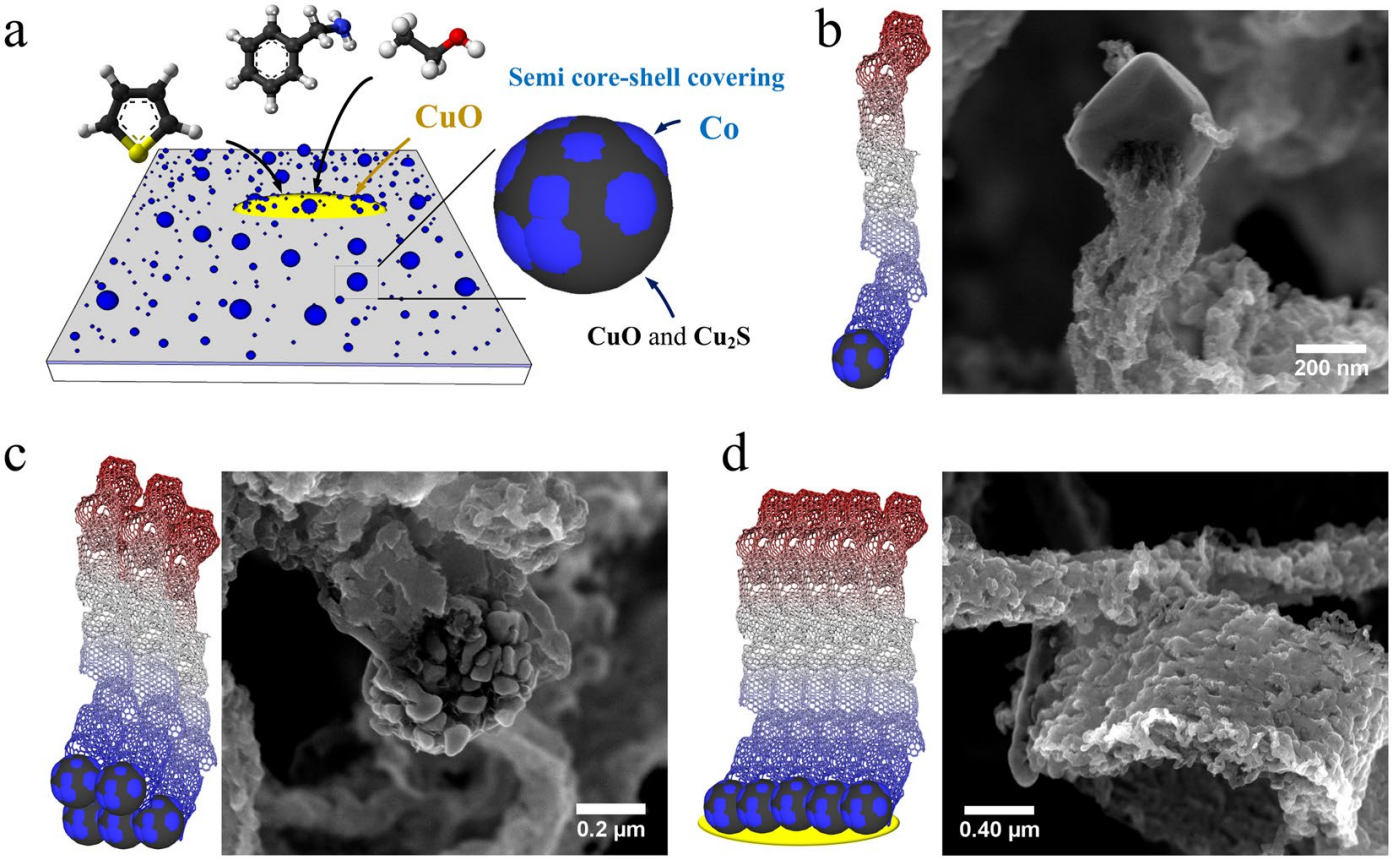
Growth mechanism of wrinkled carbon fibres and N-CBs: (**a**) at the beginning, Co nanoparticles (80–300 nm) are formed and are covered with copper, copper-oxide and copper sulphide; (**b**) at a second step, the wrinkled carbon fibres grow from the such covered Co nanoparticles; (**c**) several wrinkled carbon fibres are assembled by the agglomeration of Co nanoparticles with a garlic cloves-like shape; (**d**) the final structure is formed by the repetition of the assemblage and Co particles agglomeration.

The formation of N-CB structures is unclear, however, some experimental evidence from the characterization techniques provides clues regarding the growth mechanism. In Fig. 6, a possible growth mechanism of the N-CBs is proposed. Before turning on the sprayer, the Co/Cu film is probably transformed in Co@Cu nanoparticles, which are randomly dispersed over the substrate^50^. When the sprayer is turned on, vapour containing carbon, oxygen, and sulfur interacts with such Co@Cu nanoparticles promoting the formation of CuO and Cu_2_S zones on the Co nanoparticle surface (Fig. 6a). These zones alter the typical growth of carbon structures, but the uncovered cobalt develop the formation of complex carbon structures as shown in Fig. 6b. During the CVD experiment, these individual complex structures form agglomerates as shown in Fig. 6c. In addition, CuO nanobars are formed during the synthesis (see Fig. 4a) that serve as a nucleation zone of the N-CB morphologies (Fig. 6d). However, more experimental studies are needed to fully understand how the N-CB grow.

In conclusion, a novel method is proposed to synthesize an amazing sp^2^ carbon material with fascinating structural irregularities. This material consists of nitrogen-doped carbon belts (N-CBs) that were synthesized using the template-based chemical vapour deposition method. Cu/Co films fabricated using magnetron sputtering were used as the template. N-CBs grew when a nebulized solution containing ethanol-benzylamine-thiophene was pyrolized and passed through the reactor for 2 hours. The N-CBs grew in the zone where both tubular furnaces were joined. A closer examination of the N-CB structures revealed that these structures were formed by assembled wrinkled carbon fibres that were laterally joined. By TEM characterizations, it was demonstrated that the corrugated carbon fibres were highly defective sp^2^ carbon materials. The findings indicate production of a novel carbon material with several structural irregularities (holes, edges, corrugation, and doping) that could serve as an excellent ORR catalyst. Furthermore, this material could use as a high capacity electrode for super capacitors and lithium ion batteries in nano-devices. Other templates formed by noble metals on Fe, Co, or Ni could be used for the synthesis of exotic carbon structures.

### Method and experimental details

Cobalt-copper thin films were deposited on Si/SiO_2_ 8 cm × 8 cm square pieces that were previously cleaned, first by ultrasonic bath acetone (99.974% - Fermont), then by using ethanol (96.5% - CTR Scientific), and finally by isopropanol (99.8% - CTR Scientific). Each cleaning process lasted for 30 min. Flushing with N_2_ (Infra, 99.9995%) gas was used to rapidly dry the substrate surface. A modified Intercovamex V3 sputtering system was used to deposit cobalt and copper thin films over rotating Si/SiO_2_ substrates. At a pressure of 2 × 10^−8^ Torr argon gas was introduced into the deposition camera to produce cold plasma during the coating process at 2 × 10^−2^ Torr and 1.2 × 10^−2^ Torr for Co and Cu thin films, respectively. A DC MDX-500 power supply at 30 W was used to perform the deposition of Co thin films at a 20 nm of thickness. In the case of Cu, a pulsed DC power supply “Pinnacle plus”, was used at 15 W for 66 seconds to deposit Cu to a 2 nm of thickness^50^. The samples were stored under a nitrogen atmosphere to prevent oxidation. The production of N-CBs was conducted using a two-furnaces configuration of the AACVD method, as shown in Fig. S1 (Supplementary Information). The precursor solution was prepared with 49.75% w/w benzylamine, 49.93% w/w ethanol and 0.32% w/w thiophene as sources of nitrogen, carbon and sulphur, respectively. A quartz tube that was 1.1 m in length with one-inch internal diameter was employed for synthesis. The temperature of the furnace near the sprayer was 750 °C (furnace 1); the other furnace had a temperature of 850 °C (furnace 2). If the two furnace are not at these two temperatures the N-CB is not formed. Si/SiO_2_ substrates that were 1 cm × 1 cm and previously placed alongside the reactor were exposed to a reduction process for 5 min.; then, a flow of 2.5 L/min H_2_-Ar (5–95%) carried the nebulized precursor solution for 120 min. The samples were characterized by scanning electron microscopy (SEM) and backscattered electrons scanning electron microscopy (BSE-SEM) using a Helios Nanolab 600 Dual Beam (FIB/SEM) and a Quanta 250 (eSEM). High-resolution transmission electron microscopy (HRTEM), Z-contrast and energy dispersive X-ray spectroscopy (EDX) images were acquired using a FEI TECNAI F30 (300 keV)  microscope. X-ray diffraction patterns were obtained by a RX Advance equipment with a copper tube and λ = 1.54 Å. Raman spectroscopy studies were performed with Renishaw Micro-Raman equipment with a 532 cm^−1^ laser wave length, and the X-ray photoelectrons spectroscopy (XPS) PHI 5000 Versaprobe II equipment with an Al-Ka (1486.7 eV) source was employed to obtain concentrations of C, N, O, Co, Cu and S at 23.9 eV of pass energy. The magnetic properties were measure using a physical property measurement system (PPMS) Dynacool Quantum Design at 300 K. For the electrochemical properties a Calomel electrode in a KCl saturated solution was used as a reference electrode. The N-CBs were pulverized using a silica ball mill over 5 min and mixed in a 10% w/w solution with Nafion® (10% w/w on ethanol - Sigma), the suspension was deposited over a 2.2 mm diameter graphite electrode to be used as work electrode. The cyclic voltammetry was developed using a KCl 0.5 M solution as electrolyte, with a potential window from −1.5 V to 1.7 V at a scan rate of 100 mV/s.

## Electronic supplementary material

Supplementary Information
