# Supplementary material for: Wrinkled Nitrogen-doped Carbon Belts
Source: Sci Rep. 2018 Feb 23;8:3546. doi: 10.1038/s41598-018-21898-6 (PMC5824786; doi:10.1038/s41598-018-21898-6)
Supplement: Supplementary file 1 — Supplementary Information [file 41598_2018_21898_MOESM1_ESM.docx]

**Supplementary Information**

**Wrinkled Nitrogen-Doped Carbon Belts**

Juan L. Fajardo-Díaz, Florentino López-Urías, Emilio Muñoz-Sandoval

*Advanced Materials Division, IPICYT, Camino a la Presa San José 2055, San Luis Potosí 78216, Mexico*

**
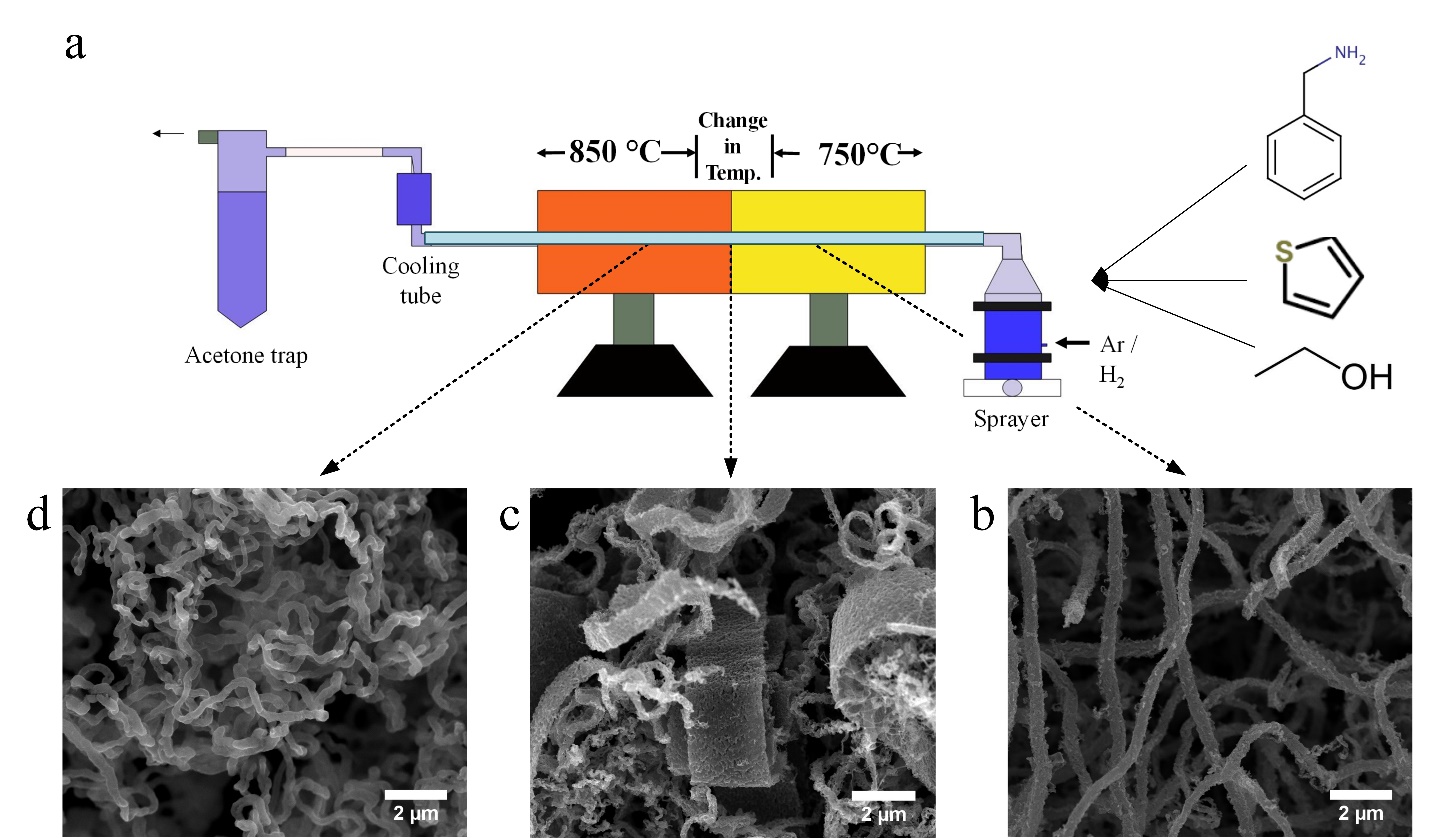
Figure S1:** Juan L. Fajardo Díaz et al.

**Figure S1:** **(a)** Schematic experimental setup of aerosol assisted chemical vapor deposition for the production of wrinkled nitrogen-doped carbon belts (N-CBs) and carbon fibers and belts. Benzylamine, thiophene, and ethanol were used as precursors. The system consists of two tubular furnaces. **(b)-(d)** SEM images showing the different carbon nanostructures collected alongside the reactor (tube quartz). N-CBs showed in **(c)** were grown where both tubular furnaces are joined.

**Figure S2**: Juan L. Fajardo-Díaz et al.


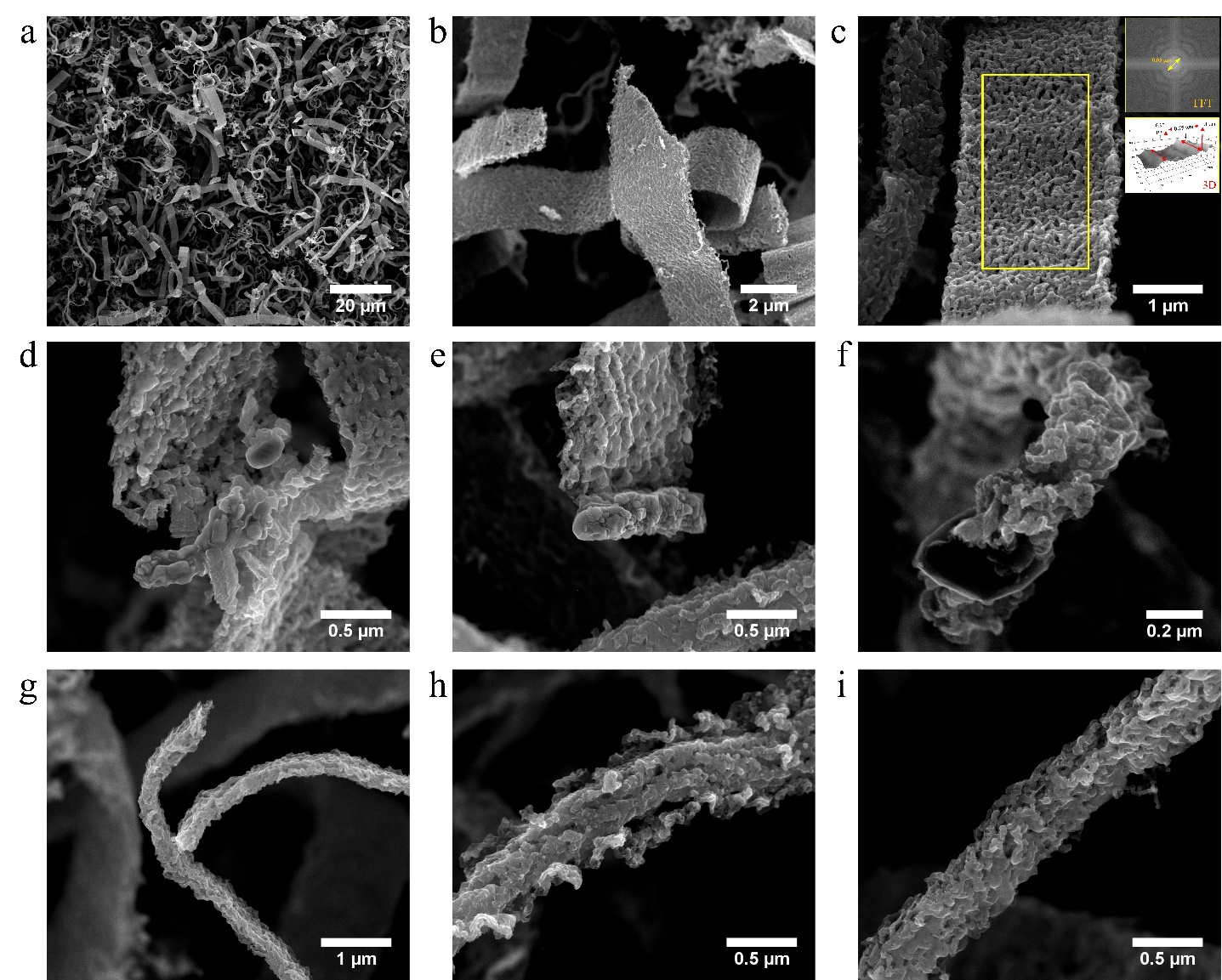


**Figure S2:** SEM images showing the N-CB structures: **(a-c)** N-CBs and wrinkled carbon fibers, **(d-e)** Particles catalyzing N-CBs and wrinkled carbon fibers, **(g-i)** wrinkled carbon fibers. The above and below insets in **(c)** are a FFT analysis and a surface representation of the image inside the yellow square, respectively.

**Figure S3:** Juan L. Fajardo-Díaz et al.


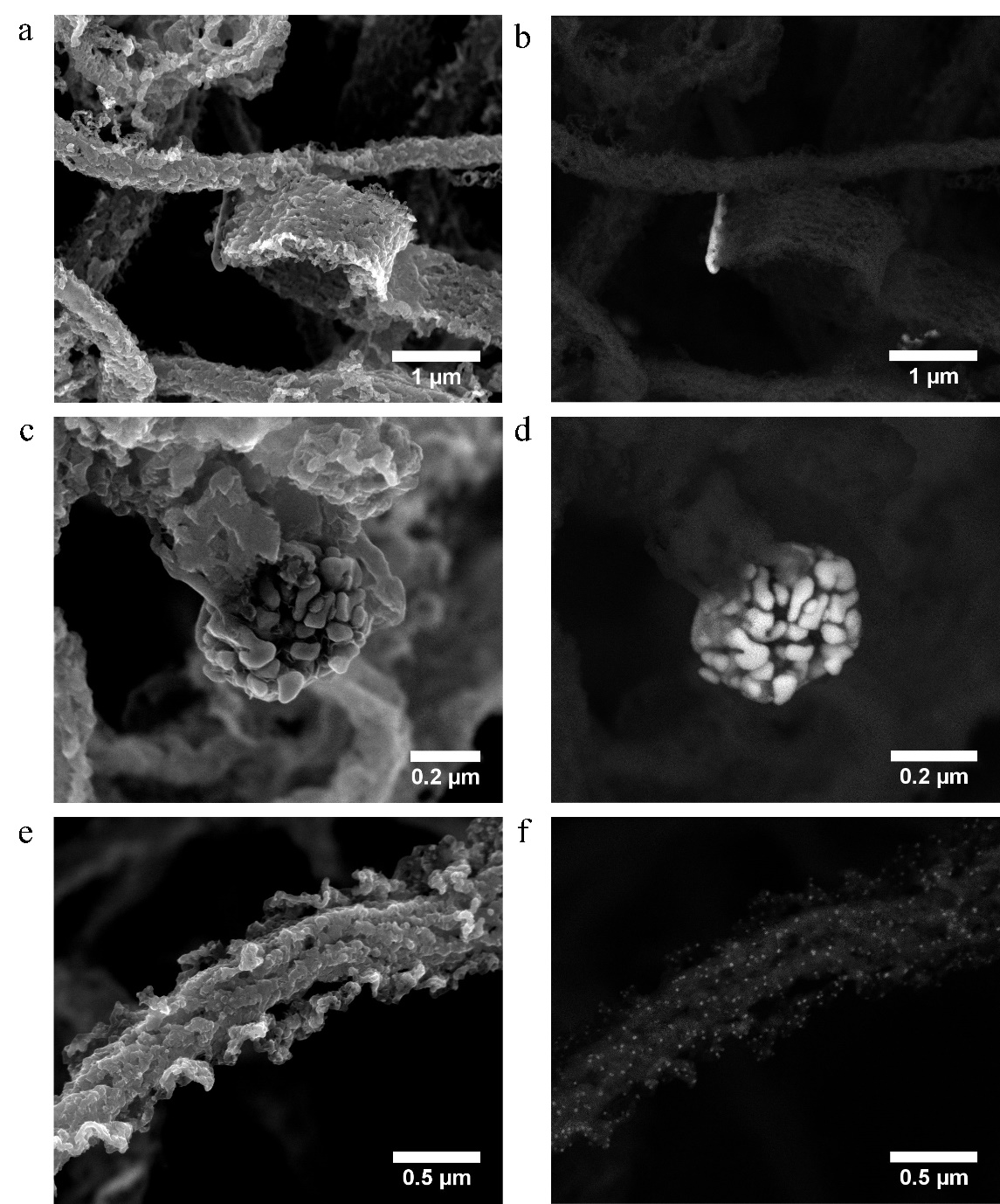


**Figure S3:** SEM images (left side images) and the corresponding backscattering electron images (right side images) showing the metallic particles inside the samples. **(a)-(b)** Catalytic metal particle placed at the tip of a N-CB. **(c)-(d)** images showing the root growth and the assembled cobalt nanoparticles form a head of garlic-like morphology. Each garlic clove (Co-nanoparticle) catalyze a wrinkled carbon fiber. **(e)-(f)** Wrinkled carbon fiber hosted copper nanoparticles on the surface.

**Figure S4:** Juan L. Fajardo-Díaz et al.

**
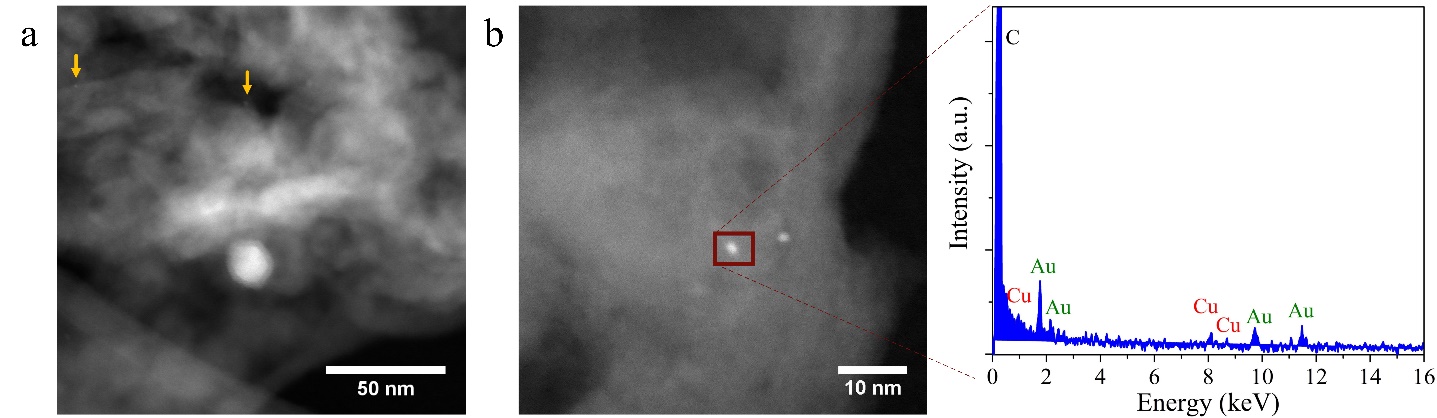
**

**Figure S4:** Z-contrast-TEM images of embedded particles into the N-CBs. **(a)** nanoparticle of around ~2 nm (yellow arrows) slightly visible and a Co-nanoparticle (~20 nm). **(b)** High resolution in Z-contrast TEM image of two particles with 2 nm diameter, and **(c)** EDS analysis confirming that the nanoparticle in the enclosed square is composed of copper.

**Figure S5:** Juan L. Fajardo-Díaz et al.


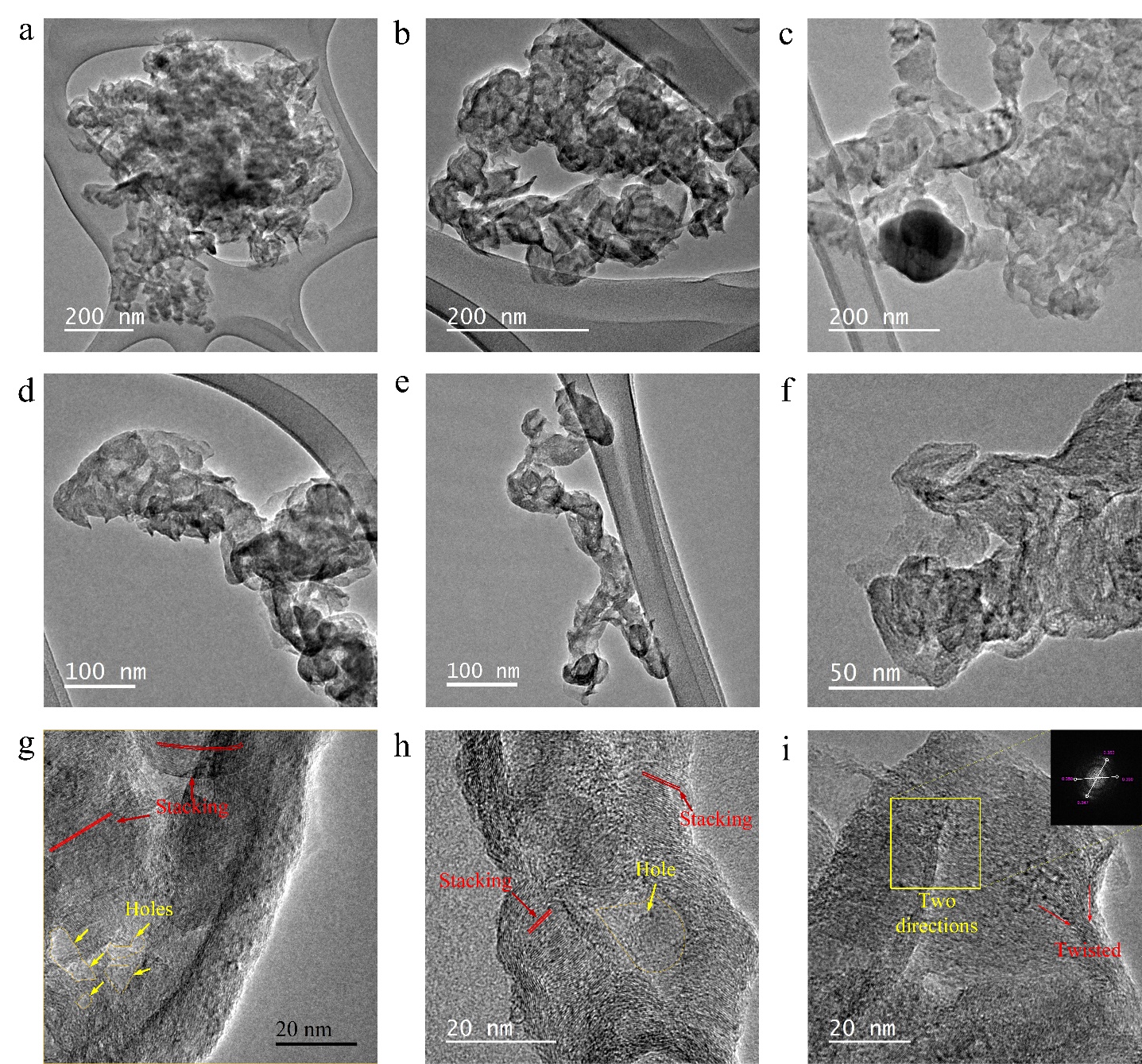


**Figure S5:** TEM and HRTEM images of wrinkled carbon fibers. **(a)-(f)** The structures grew in an irregular fashion. **(g)-(i)** Edges and holes in wrinkled carbon fibers.

**Figure S6:** Juan L. Fajardo Diaz et al.


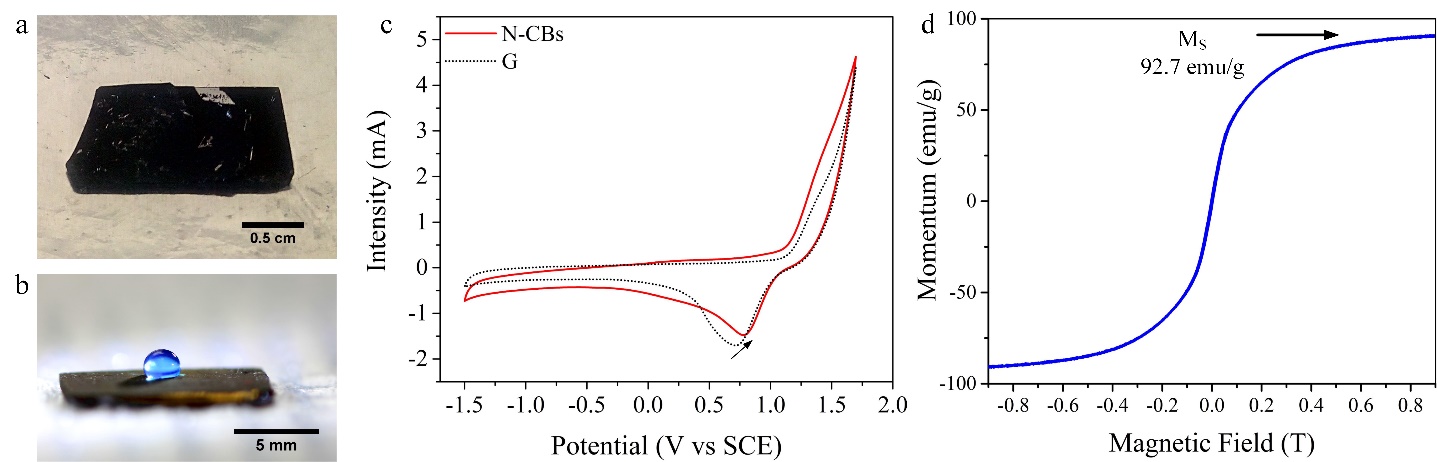


**Figure S6:** **(a)** Si substrate N-CBs on its surface. **(b)** A colored water drop over the hydrophilic substrate. **(c)** A cyclic voltammetry using a graphite electrode (G black line) and N-CBs deposited over the graphite electrode (N-CBs red line). A potential reduction is observed in the N-CB case (see black arrow). Also an increment of the ORR current is observed. **(d)** Magnetization versus applied magnetic field curve of the N-CBs showing a superparamagnetic behaviour.

**Figure S7:** Juan L. Fajardo-Díaz et al.


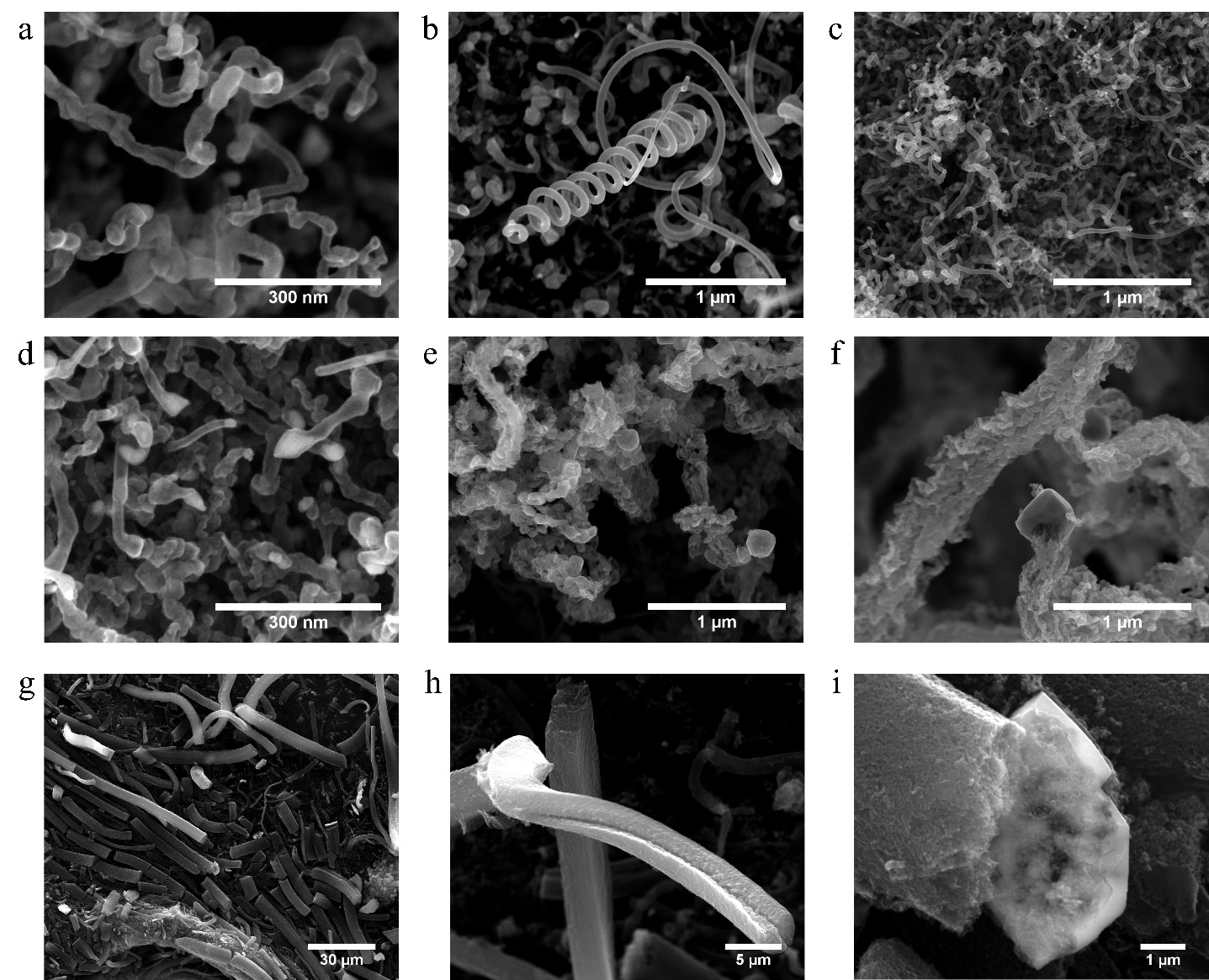


**Figure S7:** SEM images showing carbon nanostructures synthesized by varying the precursors in the CVD experiment. In all cases, the synthesis time was maintained for 30 min. Structures obtained using as precursors: **(a)** ethanol, **(b)** benzylamine, **(c)** benzylamine and ethanol in a 1:1 ratio, **(d)** ethanol-tiophene, **(e)** benzylamine-thiophene, **(f)** benzylamine-ethanol-thiophene. **(d)-(f)**, thiophene concentration is low (0.32 % of thiophene). In **(e)** and **(f),** a tip growth complex architectures is clearly observed. **(g-i)** Varying the concentration of cobalt and copper different types of N-CBs were grown using the same configuration AACVD system.

**Tables and captions**

**Table S1:** Juan L. Fajardo-Díaz et al.

| C1s | eV | FWHM | % | N1s | eV | FWHM | % | O1s | eV | FWHM | % |
| --- | --- | --- | --- | --- | --- | --- | --- | --- | --- | --- | --- |
| Carbide | 283.8 | 0.99 | 2.8 | Pyridinic | 398.6 | 1.81 | 32.6 | CuO | 528.9 | 0.77 | 1.6 |
| C=C | 284.7 | 0.85 | 59.7 | Pyrrolic | 400.1 | 1.67 | 33.7 | Cu_2_O | 530.3 | 1.33 | 9.0 |
| C-C | 285.4 | 0.60 | 11.9 | Quaternary | 401.2 | 1.51 | 17.4 | C=O | 531.3 | 1.31 | 14.0 |
| C-O | 286.0 | 0.95 | 9.5 | N-O | 402.8 | 3.08 | 16.2 | C-O | 532.5 | 1.45 | 40.8 |
| C=O | 287.0 | 1.72 | 7.2 |  |  |  |  | COO^-^ | 533.8 | 1.54 | 26.9 |
| COO^-^ | 288.3 | 3.71 | 5.5 |  |  |  |  | COOH | 534.5 | 1.88 | 7.7 |
| π−π | 291.6 | 3.57 | 3.4 |  |  |  |  |  |  |  |  |

**Table S1:** Deconvolution results of C1s, N1s, and O1s XPS spectra of nitrogen-doped carbon belts. The gravity center in eV, full width at half maximum (FWHM), and the area under the curve of the different chemical species (%). This area provides a quantitative estimation of the different chemical species in the sample. In the case of the O1s results, a high concentration of C-O species probably corresponds to phenolic or ester groups, and the presence of copper oxides.
